# Desegregating spaces: The interplay between ecological intergroup contact and GPS‐traced spatial segregation among youth in two UK cities

**DOI:** 10.1111/bjso.70043

**Published:** 2026-01-05

**Authors:** Marco Marinucci, Christoph Daniel Schaefer, Pier‐Luc Dupont, David Manley, Laura K. Taylor, Shelley McKeown Jones

**Affiliations:** ^1^ University of Milano‐Bicocca Milano Italy; ^2^ University of East Anglia Norwich UK; ^3^ Kiel University Kiel Germany; ^4^ Swansea University Wales UK; ^5^ University of Bristol Bristol UK; ^6^ University College Dublin Dublin Ireland; ^7^ University of Oxford Oxford UK

**Keywords:** ecological momentary assessment, everyday intergroup contact, GPS‐GIS analysis, socio‐spatial segregation

## Abstract

Recent advances in intergroup contact research have drawn on methods from human geography to investigate how segregation shapes, and is shaped by, everyday intergroup experiences. Emerging findings suggest that the phenomena might be reciprocally intertwined, but empirical evidence is limited and mixed. This research tested the reciprocal relationship between everyday intergroup contact and segregation using ecological momentary assessment and GPS‐GIS tracking in two segregated UK cities with youths aged 15–17. Study 1 (Belfast; *n*_participants_ = 15; *n*_interactions_ = 115; *n*_GPS‐point_ = 633) focused on Catholics–Protestants divisions, and Study 2 (Bradford; *n*_participants_ = 30; *n*_interactions_ = 334; *n*_GPS‐point_ = 2868) addressed ethnic segregation among Asian, White, and Black communities. In both studies, youths reported on social interactions throughout 6 days, while their urban mobility in outgroup spaces was tracked. In Belfast, more mixed districts predicted higher anxiety during intergroup interactions, yet, positive intergroup contact was followed by increased visits to outgroup spaces. In Bradford, mixed districts increased the likelihood (but not the quality) of intergroup contact, while the link between positive contact and subsequent outgroup space use was replicated. The findings highlight a virtuous cycle depending on contextual norms by which positive contact and desegregation practices might reinforce each other, arguably demonstrating the potential of intergroup contact for levelling urban divisions.

## INTRODUCTION

Decades of research in social psychology investigated the *contact hypothesis* that positive intergroup contact reduces prejudice (Allport, 1954). Meta‐analytic evidence based on correlational, longitudinal, experimental, and intervention studies confirmed that contact – especially under conditions of cooperation and equal status among the partners – can reduce intergroup anxiety, foster empathy, a common identity, and ultimately support social cohesion (Dovidio et al., 2003; Paluck et al., 2019; Pettigrew & Tropp, 2008; Vezzali & Stathi, 2017). However, recent longitudinal and experimental findings suggest that these effects may be more context‐dependent and variable than previously assumed (Friehs et al., 2023; Hodson & Meleady, 2024; Lowe, 2024; Meleady et al., 2025; O'Donnell et al., 2025; Sengupta et al., 2023; Shulman et al., 2024). A key research challenge lies, therefore, in understanding when, for whom, and under which socio‐spatial conditions contact reduces prejudice, situating the study of intergroup contact within the socioecological context (Paolini, Harwood, et al., 2024).

While dominant socio‐geographical approaches overlooked the socio‐psychological mechanisms underlying spatial divisions (Hinds et al., 2022), psychological research demonstrated that intergroup processes related to prejudice, stereotypes, social identity, intergroup contact, and threat are fundamental for understanding intergroup segregation (Bettencourt et al., 2019). However, empirical studies examining the nature and effects of intergroup contact in urban spaces in near‐time are limited (see Dixon et al., 2020; Keil et al., 2020). The present research integrates the *ecological momentary assessment* of near‐time intergroup contact with GPS tracking, urban demographic data, and GIS analyses to shed new light on the reciprocal influence between socio‐spatial segregation and intergroup contact. Focusing on youth from two UK contexts characterized by high ethno‐religious and interethnic tensions, Belfast and Bradford, we investigated whether (a) socio‐spatial segregation limits the opportunity for and the quality of intergroup contact and (b) the quality of previous outgroup contact predicts the use of outgroup spaces, arguably contributing to the reduction of segregation.

### Understanding socio‐spatial segregation

Socio‐spatial segregation – the physical separation of social, economic, or demographic groups – hinders social interactions and perpetuates inequalities in economic, social cohesion, and health outcomes (Liao et al., 2025; Moro et al., 2021). Sociology and urban geography have long focused on exploring *residential segregation*, namely, how different groups cluster in separate neighbourhoods (Feitosa et al., 2007). Findings on the psychosocial consequences of residential segregation are mixed, with some highlighting benefits for minorities (e.g., facilitating cultural expression and ingroup bonds; van Kempen & Bolt, 2012), and others pointing out detrimental repercussions (e.g., increased criminality and socioeconomic inequalities Oberwittler, 2007; Orfield & Lee, 2005). Beyond possible contextual differences, mixed findings may stem from methodological limitations in studying residential segregation. Residential data cannot fully capture the dynamic nature of segregation, which people might experience in multiple contexts beyond their residential districts, such as work and school neighbourhoods, and leisure spaces (Lysaght & Basten, 2003; Netto et al., 2015). Consequently, research has shifted towards analysing mobility patterns of human activities in everyday life spaces. The study of *activity space segregation* examines individuals' travel behaviours and visited locations to assess the co‐presence of people from different groups in the same space at the same time (Müürisepp et al., 2022). This approach has been enabled by technological advances and big human mobility data, including GPS tracking and geotagged social media posts (Liao et al., 2025). These advances have allowed exploring the demographic make‐up of visitors of urban spaces (e.g., income, ethnicity, and gender, Moro et al., 2021) as well as individuals' co‐presence with people from different backgrounds (Wu et al., 2023). Findings highlight that social groups' mobility patterns are often highly separated by demographic factors like income (Moro et al., 2021; Nilforoshan et al., 2023), religion (Dixon et al., 2020; Greenberg Raanan & Shoval, 2014), migration status (Gao et al., 2021; Ta et al., 2021), and ethnicity (Järv et al., 2015).

While the focus on activity space has advanced the study of socio‐spatial segregation, mere co‐presence in the same space and time is not a sufficient indicator of integration, as physical proximity does not ensure social interaction (Liao et al., 2025; Zhou & Cheng, 2019). Although some studies linked spatial co‐presence and intergroup interactions (Blumenstock & Fratamico, 2013), evidence suggests that people from different groups tend to live *parallel lives*: sharing spaces without interacting (Bettencourt et al., 2019; Dixon & Durrheim, 2003; Valentine, 2008). While notable previous research has examined intergroup contact within dynamics of socio‐spatial segregation (see the Belfast Mobility Project https://belfastmobilityproject.org/; Dixon et al., 2020), whether co‐presence leads to meaningful social interactions remains an open question that cannot be addressed by a geographical perspective based on mobility data alone (Cagney et al., 2020; Liao et al., 2025), but could benefit from an interdisciplinary bridge with socio‐psychological research.

### Reciprocal dynamics between intergroup contact and segregation

The literature linking intergroup contact and segregation suggests a bidirectional relationship between the two. Evidence on how the geographical context shapes intergroup contact is mixed. Some found that neighbourhood segregation can impede intergroup contact (Van Der Laan Bouma‐Doff, 2007) and that residential mixing can further increase interethnic tensions and prejudice towards minorities via negative intergroup interactions (Kotzur & Wagner, 2021). While others showed that living in mixed neighbourhoods reduced ingroup bias and social distance towards ethnic minorities (Schmid et al., 2013), leading to engaging in more positive interethnic interactions that foster neighbourhood trust (Schmid et al., 2014). These findings point to a paradoxical scenario where positive and negative intergroup dynamics coexist, suggesting that desegregation does not automatically translate into meaningful integration (Dixon et al., 2023). Indeed, in real‐life settings – especially those without institutional support – intergroup contact may imply negative experiences, outweighing the potential benefits of positive contact (Kotzur & Wagner, 2021; Marinucci et al., 2022; Paolini, Gibbs, et al., 2024).

Conversely, socio‐spatial segregation might be grounded in intergroup processes of resegregation, namely, people's tendency to stay within ingroup spaces and avoid outgroup spaces and interactions (McKeown & Dixon, 2017). Interethnic prejudice might drive individual preferences for ethnic ingroup neighbours, contributing to residential segregation (Van Der Laan Bouma‐Doff, 2016). Also, preferential segregation might occur on a *micro‐ecological* level, where social practices sustain intergroup divisions even in desegregated contexts (Dixon et al., 2008; McKeown et al., 2012). This has been evidenced in contexts including school classrooms (McKeown et al., 2016, 2017) and public beaches (Dixon & Durrheim, 2003) and is suggested to be because of stereotypes and negative attitudes, social identity boundaries, and feelings of threat in intergroup interactions (Bettencourt et al., 2019).

GPS tracking studies further support the micro‐ecological findings on activity space segregation. Greenberg Raanan and Shoval (2014) showed that Palestinian Muslim and Jewish women avoided Jerusalem districts perceived to belong to the other group. Similarly, Dixon et al. (2020) showed that Catholic and Protestant residents in Belfast seldom enter traditionally outgroup spaces. Notably, this study integrated a retrospective survey of intergroup contact with GPS mobility behaviours, showing that positive (negative) contact had a positive (negative) indirect effect on the time spent in outgroup zones via reduced safety threat. Besides, the survey findings show that positive intergroup contact was associated with higher intentions to use outgroup spaces, with the effect being mediated by reduced intergroup anxiety, perceived realistic threat to personal safety, and symbolic threat to cultural traditions. In another study in South Africa, Dixon et al. (2023) provided additional self‐reported correlational findings that positive intergroup contact of Indians with the Black African community had an indirect negative effect on Indians' avoidance of the outgroup districts via reduced safety threat.

Overall, the research bridging socio‐spatial segregation and intergroup contact offers both challenges and opportunities. While there is consistent evidence for segregation in shared spaces (Bettencourt et al., 2019; Dixon et al., 2020; Dixon & Durrheim, 2003; McKeown et al., 2016), it is not clear whether and how segregation shapes intergroup contact opportunities (Liao et al., 2025) with inconsistent findings suggesting that residential mixing can increase both positive and negative contact, leading to reduced or increased prejudice, respectively (Kotzur & Wagner, 2021; Schmid et al., 2014). Likewise, it is not clear if contact can contribute to reducing segregation. Preliminary findings suggest that positive contact is associated with willingness to use outgroup spaces by reducing the psychosocial factors driving preferential segregation (e.g., threat; Dixon et al., 2020, 2023). However, this evidence is based on retrospective, correlational studies that have not captured actual or near‐time contact in real‐life settings.

The present research sought to address these gaps by using a smartphone‐based app to assess near‐time intergroup contact in everyday life, combined with a GPS tracking system measuring where the contact occurred and the spaces participants visited in the near‐time afterward (Keil et al., 2020). Responding to calls to integrate human mobility into psychological science (Hinds et al., 2022), we investigated the nature of youth's everyday intergroup contact, how residential segregation shapes intergroup contact, and the potential connection between everyday intergroup contact and youth mobility behaviours in outgroup spaces. We focused on youth from two different divided societies, given the importance of adolescence for sustaining the development of long‐term positive attitudes and intergroup relationships, driving social cohesion of future societies (Tropp et al., 2019). To the best of our knowledge, limited previous studies have investigated youths' activity space use in divided intergroup contexts through the lens of intergroup contact, making this an open and unexplored area of research that we aim to initiate.

## THE PRESENT RESEARCH

We conducted two event‐contingent *ecological momentary assessment* (Shiffman et al., 2008) studies investigating how interactions with ingroup and outgroup members shape youth mobility behaviours in ingroup and outgroup urban spaces. First, we explored the nature of near‐time youths' everyday intergroup interactions and whether the proportion of outgroup members in a district influences the likelihood of interacting with outgroup members and the quality of the interactions. We then tested the hypothesis that more positive contacts with outgroup members would be associated with subsequent visits to urban areas with a higher proportion of outgroup members. As a discriminant validity stress test (Rönkkö & Cho, 2020), we hypothesized that more positive contact with ingroup members would not lead to youth frequenting urban areas with a higher outgroup prevalence.

Study 1 was conducted among youth in Belfast (Northern Ireland), focusing on ethno‐religious relations between Catholics and Protestants; Study 2 was conducted among youth in Bradford (England) in the context of intergroup relations along ethnic divisions between White, Asian, and Black people. Both studies followed the same method, sampling, and analytical procedure, differing only in terms of the samples and the context.^1^

### Research contexts

Both Belfast and Bradford are imbued with past and present ethno‐religious tensions. Belfast continues to be affected by the legacy of the ‘troubles’, a conflict between those who wanted reunification with Ireland, typically Catholic/Irish/Nationalist, and those who wished to remain part of the UK (Cairns & Darby, 1998), typically Protestant/British/Unionist. Although the peace process began in 1998, religious tensions and violence have remained (Taylor et al., 2016), and the city is facing an intensification of urban segregation and division (Murtagh et al., 2024). *Peace walls* – barriers dividing Catholic from Protestant communities – spread across many areas of Belfast, with their size increasing in the post‐conflict city (Huck et al., 2019).

While Bradford lacks a history of political violence like that of Northern Ireland, it is a highly diverse city marked by interethnic tensions (Miah et al., 2020). According to the 2021 England and Wales census, the population is predominantly White/White British (56.7%) and Asian/Asian British (32.1%), with 2% identifying as Black. In 2001, the city experienced riots linked to ethnic tensions between the White and Asian communities (Waddington, 2010). Bradford is also among the British cities that are facing the highest level of residential segregation, with districts that are mainly inhabited by ethnic minorities and increasingly less inhabited by the White British majority (Lan et al., 2020).

## METHODS

### Participants

Youths were recruited through educational and community‐based organizations in Belfast and Bradford. For Belfast Study 1, we excluded one participant of mixed Catholic–Protestant religion and one participant whose GPS and interaction positions corresponded to his home position throughout the 5 days of data collection. The remaining sample consisted of 15 adolescents aged 16–17 years (12 females, 3 males; 10 Catholics, 5 Protestants). Overall, participants reported a total number of 115 social interactions and 633 valid GPS records over a median data collection period of 3 days (range = 2–6 days).

In Bradford Study 2, we excluded three participants of mixed ethnicity, and the final sample consisted of 30 adolescents aged 16–17 years (19 females, 11 males), with White (*n* = 18), Asian (*n* = 7), and Black (*n* = 5) ethnicity. Overall, they reported 334 social interactions and 2868 valid GPS records over a median data collection period of 4.5 days (range = 2–6 days).

Participants of mixed religious or ethnic groups were excluded as it was not possible to determine unambiguously their ingroup or outgroup of reference.

### Procedure

The research received ethical approval from the University of Bristol. All research data, analysis codes, and Appendix S1 are available via this OSF link (https://osf.io/vt4u8/overview?view_only=467e3c2034414f2a90c2b0b0e19744e2).

Data were collected using a bespoke version of the *Contact Logger* smartphone application (Keil et al., 2020; see https://contactlogger.app/). The app was designed to capture contact events in near‐time. It enabled the recording of key characteristics of each interaction (e.g., type of situation, perceived quality) and the geographic location. An extra feature for the present research was that the app allowed participants to activate GPS tracking.

In both studies, participation included a 1‐h introduction workshop where youth were informed about the project and the purpose of the research and instructed on how to use the app, followed by 1 week of data collection. Participants were informed that the project was broadly interested in understanding more about the factors that influence intergroup interactions in urban spaces, without revealing the explicit hypotheses about spatial segregation. Following the opportunity to ask questions about the project and what they would be expected to do if they were interested in taking part, youth were given an information sheet and consent form. After providing written consent, they were provided with a pre‐configured mobile phone with the *Contact Logger* app installed. They were instructed to use the app for the next 6 days to report each social interaction with anyone shortly after it occurred (except during school/college or work time, which were reported after school/work time). Each entry asked them to include details about the interaction partner, the location, and their overall interaction experience. Participants were also asked to activate the background GPS tracking by tapping an icon on the app every time they left home or college/work and during their leisure time. The GPS tracking function could be turned on or off independently of their interaction logging.

At the end of the project, participants chose between a £100 Love2Shop or Amazon voucher or receiving the phone they used for the research as compensation for their time devoted to the research.

### Measures

Prior to using the app, youth completed a brief demographic form reporting their age, gender, and community/ethno‐religious background. This information was used to determine their group membership and which groups represented an outgroup when reporting interactions in the app.

#### Social interactions

Participants used the app to answer a series of single items assessing the setting (e.g., home, food, and drink outlet) and situation (indoor vs. outdoor) of the contact, how long the interaction lasted, and how formal (1 = very casual, 5 = very meaningful) the interaction was. Participants also reported the type of relationship with the person they interacted with (e.g., friend, stranger), the interaction partner's gender, age group, ethnicity, religion, and perceived group typicality (1 = not typical at all, 5 = very typical), and the interaction's quality in terms of discomfort (1 = not at all uncomfortable, 5 = very uncomfortable) and quality (1 = very negative, 3 = neutral, 5 = very positive). Lastly, participants were asked to pinpoint on a map the exact position where the interaction occurred (see the Appendix S1 for full details).

#### GPS tracking

Participants could voluntarily turn on/off the GPS background tracking by activating/deactivating an icon on the *Contact Logger* app. The app recorded latitude, longitude, date, and exact time every 10 s. The app also recorded the accuracy in metres of the GPS points. In both studies, we excluded all the GPS points with an accuracy higher than 30 metres (Study 1 = 105, Study 2 = 481 excluded GPS points).

### Analytical procedure

#### Scoring of district outgroup prevalence

Participants' GPS positions within an urban district were associated with a score representing the proportion of outgroup members in that district. This allowed us to transform the GPS data into a measure quantifying the outgroup prevalence of the districts where participants have been.

To do so, we used the publicly available 2021 census data in Northern Ireland (https://www.nisra.gov.uk/statistics/census/2021‐census) for Study 1, and in England and Wales (https://www.ons.gov.uk/census) for Study 2. Both sources make all census estimates (e.g., religious or ethnic prevalence in all districts) and geographical outputs (shapefiles codifying the digital geographical boundaries of the districts) available for download. In each case, we downloaded the lowest tier available geographical units delineating districts similar in terms of population size and housing characteristics, namely, *Data Zones* for Northern Ireland (including on average 500 persons; see NISRA, 2023) and *Output Areas* for England (including between 100 and 625 persons). We also downloaded *Data Zones*' estimates about persons' religion (or religion of upbringing if religion was unavailable; see NISRA, 2022) and *Output Areas*' data about persons' ethnicity.

We merged the GPS data with the geographical outputs and the associated religion and ethnicity estimates using *R* (version 4.3.3; R Core Team, 2024) and package *sf* (Pebesma & Bivand, 2023). We computed outgroup prevalence for each GPS point based on the district where the GPS position fell. The exclusion of GPS points with low accuracy ensured the quality of the match between the GPS points and the district. In Study 1, we computed outgroup prevalence for Catholic (Protestant) participants as 100 *minus* the percentage of Catholic (Protestant) prevalence in the district. Similarly, in Study 2, we computed outgroup prevalence as 100 *minus* participants' ethnic group prevalence in percentage.

#### Scoring of the main analyses' dependent variable

The main dependent variable was the average score of the outgroup prevalence of the districts where participants had been following logging an ingroup or outgroup contact. If participants reported multiple interactions in a day, the score reflected the average outgroup prevalence of districts visited before the next interaction was reported. Otherwise, it reflected the average outgroup prevalence of districts visited from the time of the reported interaction until the end of the day. We considered only GPS points recorded after an interaction with either ingroup or outgroup members, excluding all other GPS points and the location of the interaction itself. Higher scores indicated greater presence in districts with a higher proportion of outgroup members in the near‐time after each contact within a day, or between contacts in a day if more interactions were reported. Two .*html* maps plotting the interactions with the ingroup and outgroup, the GPS tracking following each interaction, and the districts' ethno‐religious or ethnic prevalence are available on the OSF.

#### Statistical analyses

We first explored descriptive patterns of interactions and districts visited. We then conducted preliminary analyses to investigate whether interaction quality differed by ingroup vs. outgroup interactions and by the districts' outgroup proportion. Finally, we tested the main hypothesis that more positive contact with the outgroup (vs. the ingroup) would be associated with youth frequenting districts with higher outgroup proportions by testing a mixed moderation model using the *lmerTest* (Kuznetsova et al., 2017) and *interactions* (Long, 2019) packages; effect sizes were computed with the *EMAtools* package (Kleiman, 2017). We ran a three‐level multilevel model, with a fixed slope and random intercepts, with the levels being (1) hours within days within participants, (2) days within participants, and (3) participants (see the *Main Results* section for details). We included random intercepts at the participant, day, and hour levels. Interaction valence and discomfort were the main predictors, and the group with whom the interaction occurred served as a moderator.

## STUDY 1 RESULTS

### Descriptive results

#### Interactions' characteristics

Table 1 reports the characteristics of the interactions. Most occurred with individuals rather than groups, during leisure time activities, most frequently with White adults, followed by teenagers. Interaction partners were mostly friends, strangers, or acquaintances, and on average, the interactions were casual (*M* = 2.1, SD = 1.07). The median interaction duration was 6 min, ranging from 2 s to 14 h. The interactions equally occurred with females and males, of same‐ or cross‐genders, and with Catholics or Protestants. Interaction partners were perceived as somewhat typical (*M* = 2.68, SD = 1.59). Except for two negative and six uncomfortable interactions, interactions were positive (*M* = 4.16, SD = 0.83) and comfortable (*M* = 1.43, SD = 0.81). Interactions occurred over 53 different zones in Belfast and surrounding areas with, on average, 49.04% Catholic proportion (SD = 33.08), 35.49% Protestant proportion (SD = 26.67), and 50.91% outgroup residential proportion (SD = 29.89). Participants reported more frequent interactions with ingroup than outgroup members.

**TABLE 1:** Descriptive characteristics of Study 1 interactions.

Total interactions (*n* = 115)		
Situation (71.4% inside, 28.6% outside) (89.5% dyadic, 10.5% group)	23.7% leisure time (shopping, café, clubs) 18.4% walking 17.5% home 16.6% social events	9.6% online 6.1% work 5.3% transport 2.6% other
Relationship (57.1% superficial, 42.9% close)	34.2% friend 16.7% strangers 14.9% acquaintance 12.3% customer/colleague	9.7% relative 5.3% service clerk 5.3% other 1.8% partner
Ethnicity	92.6% White 3.7% mixed/unsure 1.9% other	0.9% Black (*n* = 1) 0.9% Asian (*n* = 1)
Age group	53.0% adult 34.8% teenager	10.4% older adult 1.7% child
Gender	51.4% female 48.6% male	50.5% same gender 49.5% cross‐gender
Religion	38.0% Catholic 36.1% Protestant	25.9% mixed/unsure/other
Group	Ingroup (*n* = 59) Outgroup (*n* = 21)	Missing (*n* = 35)

#### GPS tracking and urban districts

Overall, the 633 GPS records covered 51 Belfast and surrounding districts, inhabited by 51.2% of Catholics (SD = 34.2) and 34.5% of Protestants (SD = 27.7; Outgroup: *M* = 50.70%, SD = 32.1). From the full sample, we considered 382 GPS points to compute the dependent variable. The 382 GPS points occurred in 44 data zones and were grouped into 74 tracking blocks, corresponding to the available number of GPS tracking chunks that were recorded after an interaction. On average, tracking blocks included 5.16 GPS points (SD = 5.85, range = 1–35). Most tracking blocks spanned ~40 s (median = 37.2 s). However, the timespan varied largely between tracking blocks, ranging from 10 s to 10.4 h, covering on average 37 min (SD = 102.6 min). For most participants, the short‐spanning GPS tracking indicated a snapshot of where participants were after they logged an interaction. However, the tracking blocks spanning longer time periods provided a broad map of where participants passed through after the contact. In such cases, the data reflect moments when participants intermittently activated location tracking, resulting in partial traces of their mobility following the reported interaction. To limit the risks of capturing unrelated movements that are no longer meaningfully connected to the social interaction of interest, we retained for analysis only tracking blocks shorter than 4 h in the main analysis, excluding those exceeding this duration (*n* = 3).

### Preliminary findings

#### Quality of interactions

*T*‐Tests showed that valence did not differ between ingroup and outgroup interactions (*t*(78) = 1.29, *p* = .203), whereas discomfort was higher in outgroup (*M* = 1.76) than ingroup (*M* = 1.22) interactions (*t*(23.8) = −2.18, *p* = .040, Cohen's *d* = 0.75). Closer inspection showed only one negative outgroup contact. One ingroup and three outgroup interactions were reported as uncomfortable.

#### District outgroup prevalence and interactions

A logistic regression showed that district outgroup prevalence did not predict the likelihood of outgroup compared with ingroup interactions (OR = 1.014, 95% CI [0.997–1.032] *p* = .121). A mixed‐model regression controlling for between‐participants difference in average interactions' valence (random intercept) showed that district outgroup prevalence did not predict the valence of the interaction (*b* = 0.00, *p* = .308), and that the effect was the same for ingroup and outgroup interactions (*b* = 0.00, *p* = .960). By contrast, outgroup prevalence predicted discomfort in interactions differently for ingroup and outgroup interactions (exp(b) = 1.007, *p* = .048): a higher outgroup prevalence was associated with higher discomfort in outgroup (exp(b) = 1.007, 95% CI [1.001–1.013], *p* = .017) but not ingroup (exp(b) = 1.000, 95% CI [0.9965–1.004], *p* = .877) interactions.^2^ Overall, this indicates that while district outgroup prevalence did not influence the positivity of ingroup and outgroup interactions, outgroup interactions were more likely to be experienced as uncomfortable in districts with higher outgroup presence.

### Main results

The GPS positions – and the related outgroup prevalence of their district – might vary, on average, (a) between participants (i.e., different average outgroup proportions of the districts visited by each participant due to different mobility habits – for example, home location); (b) between days of data collection for each participant (i.e., different average outgroup proportions of the districts visited on different days– for instance, between school weekdays and free weekends); (c) between hours of the same day (i.e., different average outgroup proportions of the districts visited at different hours, as participants might be in districts with different outgroup prevalence in different moments of the day‐ for instance, in the morning in one's house district, during the day in their school district). This nested random intercept allows isolating whether hour‐level fluctuations in positive intergroup contact predict subsequent fluctuations in the use of districts with higher outgroup prevalence, net of stable person‐specific and routine patterns in spatial mobility.

To account for the nested structure, we compared three different null models with random intercepts at (a) the participant level, (b) the day within the participant level, and (c) the hour within the day within the participant level. The likelihood ratio test and Chi‐square difference test suggested that model c had a significantly better fit both compared with model b (Δχ^2^(1) = 10.56, *p* = .001) and model a (Δχ^2^(2) = 10.57, *p* = .005). In the final model, we added within‐cluster predictors: the previous interactions discomfort and valence moderated by whether the group of interaction partner (ingroup or outgroup).

To ensure that the observed effect was not biased by the single negative outgroup interaction recorded, we excluded this from the analysis. The results showed a significant interaction between valence and the group with whom the interaction occurred (Table 2).

**TABLE 2:** Main results of Study 1.

Predictors	Estimates	95% CI	*p*	Cohen's *d*
Fixed effects				
(Intercept)	46.12	4.20 to 87.36	.**040**	
Discomfort	4.64	−4.50 to 13.62	.336	0.27
Group	−102.66	−182.19 to −17.65	.**020**	−0.66
Valence	−2.91	−10.70 to 5.07	.490	−0.20
Discomfort × Group	6.76	−8.01 to 21.47	.388	0.24
Valence × Group	20.93	4.49 to 36.19	.**014**	0.71
*σ* ^2^	145.6			
*τ*_00_: h/day/subject Day/subject Subject	218.1 0 565.2			
*N* observations *N* (h/day/subject)	70 (17/6/14)			

*Note*: *σ* ^2^ = residual variance; *τ*_00_ = random intercept variance. Statistically significant effects are highlighted in bold.

**FIGURE 1:**
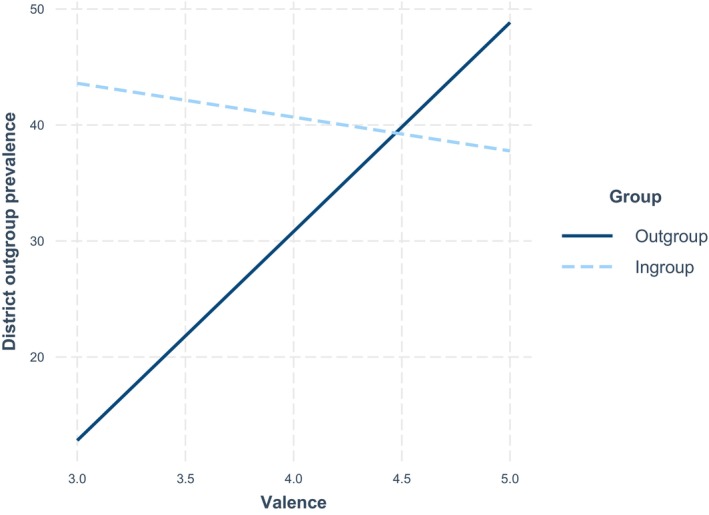
Interaction effect of contact quality by group – Study 1.

Simple slope analyses highlighted that, for interactions with ingroup members, the valence of the previous interaction did not influence youth's presence in districts with higher outgroup prevalence (*b* = −2.91, *p* = .490); by contrast, more positive outgroup interactions was associated with youth frequentation of districts with higher outgroup prevalence after the interaction (*b* = 18.01, *p* = .017). Discomfort was not associated with the district that youths frequented after the interaction for either ingroup or outgroup interactions. Figure 1 depicts the interaction effects.

**FIGURE 2:**
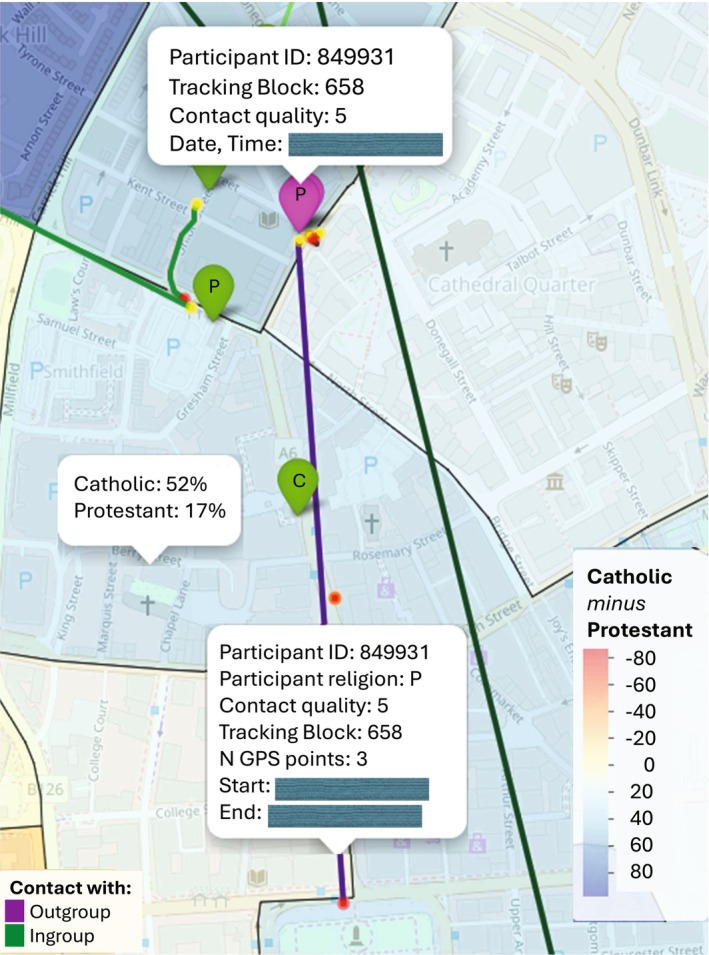
A qualitative example of the Study 1 findings in the Belfast map. Date and time information is blurred to protect the participant's anonymity.

Figure 2 shows a screenshot from the dynamic Belfast .*html* map illustrating the main findings. The purple marker indicates the position of a positive interaction reported by a Protestant participant with a Catholic friend, which took place during a study session at the Belfast Central Library. After the contact, the GPS records showed that the participant had moved to a Catholic‐majority district (blue‐shaded areas) for approximately 36 min (the purple track line), concluding the track at the Belfast City Hall Grounds.

## STUDY 2

### Descriptive results

#### Interactions’ characteristics

Table 3 reports the interaction characteristics for Study 2. The descriptive characteristics of the interactions were similar to those of Study 1. A noticeable difference is that Study 2 participants also reported interactions that occurred in their school/college setting, which could also explain why the median duration of Study 2's interactions was longer (15 min, ranging from 1 s to 18 h). As in Study 1, the interactions were perceived as casual (*M* = 2.14, SD = 1.25), and the interactions’ partners as somewhat typical of their group (*M* = 3.34, SD = 1.48). Like Study 1, almost all interactions were neutral or positive (9 negative; *M* = 4.22, SD = 0.84) and comfortable (six uncomfortable; *M* = 1.32, SD = 0.67). Interactions occurred over 88 Bradford zones and surrounding areas with, on average, 46.73% White proportion (SD = 24.84), 34.19% Asian proportion (SD = 25.70), 7.24% Black proportion (SD = 6.73), and 50.80% outgroup proportion (SD = 27.47).

**TABLE 3:** Descriptive characteristics of Study 2 interactions.

Total interactions (*n* = 334)		
Situation (74.5% inside, 25.5% outside) (72.4% dyadic, 27.6% group)	16.9% leisure time (shopping, café, clubs) 16.3% work 16.0% social events 13.8% walking 12.3% online	8.6% transport 7.1% home 4.6% school 2.5% worship place 1.8% other
Relationship (51.2% superficial, 48.8% close)	42.0% friend 14.4% acquaintance 11.7% strangers 11.7% customer/colleague/teacher	9.0% service clerk 5.1% partner 3.3% relative 1.5% other 1.2% neighbour
Ethnicity^a^	45.4% White 30.2% Asian 5.7% Black	16.1% mixed/not sure 2.5% other
Age group	63.4% teenager 32.5% adult	3.5% older adult 0.7% child (*n* = 2)
Gender	57.8% female 42.2% male	65.0% same gender 35.0% cross‐gender
Religion	60.3% Muslim 25.3% other 8.2% Protestant	1.3% Hindu 0.7% Buddhist (*n* = 2) 0.7% Sikh (*n* = 2)
Group	Ingroup (*n* = 159) Outgroup (*n* = 97)	Missing (*n* = 78)

^a^ Originally, the app assessed the ethnicity of interaction partners along 16 ethnic categories. We recoded those into the three major ethnic groups following the 2021 England and Wales CENSUS categorization (see the Appendix S1).

#### GPS tracking and urban districts

Overall, the 2868 GPS records covered 108 Bradford and surrounding districts, inhabited by 48.52% of White (SD = 23.56), 32.55% of Asian (SD = 24.07), and 7.15% of Black people (SD = 6.4; Outgroup: *M* = 55.39%, SD = 27.08). From the full GPS tracking sample, we considered 1778 GPS points to compute the dependent variable. This subset of GPS points occurred in 94 data zones and were grouped into 234 tracking blocks (i.e., the number of GPS tracking chunks recorded after ingroup or outgroup interactions). On average, tracking blocks included 7.6 GPS points (SD = 7.19, range = 1–48). The average time length of the tracking blocks was 39.7 min (SD = 90.07), ranging from 10 s to 9.2 h, with most tracking blocks covering ~1 min (median = 1.2 min). As in Study 1, we excluded from the main analyses 10 tracking blocks spanning more than 4 h.

### Preliminary findings

#### Quality of interactions

Results from a *t*‐test showed that valence (*t*(254) = 0.19, *p* = .847) and discomfort (*t*(254) = 1.50, *p* = .135) did not differ between ingroup and outgroup interactions. Participants reported two outgroup and five ingroup negative interactions. They also reported four ingroup and one outgroup uncomfortable interactions.

#### District outgroup prevalence and interactions

Unlike in Study 1, in Study 2, district outgroup prevalence was significantly associated with the likelihood of interacting with outgroup compared with ingroup members (OR = 1.026, 95% CI [1.015–1.037], *p <* .001). Controlling for between‐participants differences (random intercept), district outgroup prevalence did not predict interaction valence (*b* = 0.00, *p* = .984), and the effect was the same for ingroup and outgroup contact (*b* = 0.00, *p* = .884). Similarly, district outgroup prevalence had no effect on interaction discomfort (*b* = 0.00, *p =* .824), and this was the same for ingroup and outgroup contacts (*b* = 0.00, *p* = .806). The proportion of outgroup members in the district did not influence how positive or comfortable the outgroup contact was.

### Main results

Following Study 1, we compared three different null models to account for the data's nested structure, testing the random intercept of the district outgroup prevalence at (a) the participant level, (b) the day within the participant level, and (c) the hour within the day within the participant level. The likelihood ratio test and chi‐square difference test suggested that model c had a significantly better fit both compared with model b (Δχ^2^(1) = 34.60, *p <* .001) and model a (Δχ^2^(2) = 112.20, *p <* .001). The final model included interaction discomfort and valence, moderated by the interaction partner group membership (ingroup vs. outgroup) as the within‐cluster predictors.

As in Study 1, the negative interactions were excluded from the analyses. The results replicated Study 1 and showed a significant interaction between the valence and the group with whom the contact occurred (Table 4).

**TABLE 4:** Main results of Study 2.

Predictors	Estimates	95% CI	*p*	Cohen's *d*
Fixed effects				
(Intercept)	59.02	44.62 to 73.44	**<.001**	
Discomfort	−4.83	−7.97 to −1.83	.**003**	−0.62
Group	−25.33	−58.57 to −1.88	.**038**	−0.43
Valence	−0.45	−3.00 to 2.11	.735	−0.07
Discomfort × Group	2.04	−4.21 to 8.25	.529	0.12
Valence × Group	6.40	1.62 to 11.15	.**011**	0.53
*σ* ^2^	40.6			
*τ*_00_: h/day/subject Day/subject Subject	256.4 116.8 393.3			
*N* Observation *N* (h/day/subject)	219 (19/6/30)			

*Note*: *σ* ^2^ = residual variance; *τ*_00_ = random intercept variance. Statistically significant effects are highlighted in bold.

**FIGURE 3:**
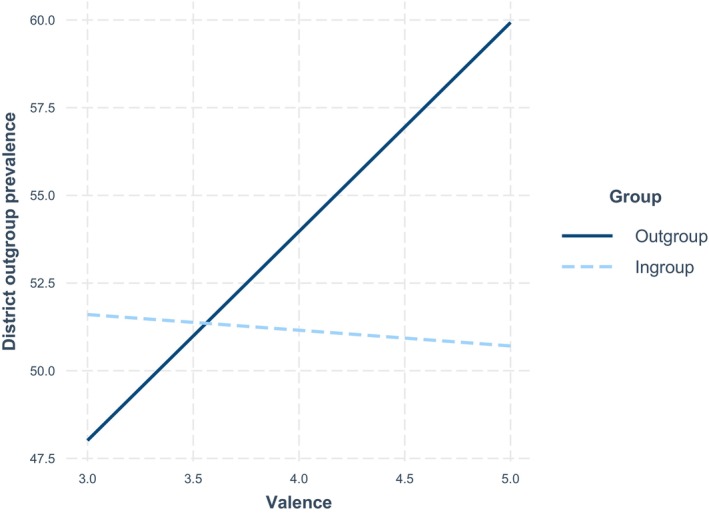
Interaction effect of contact quality by group – Study 2.

Simple slope analyses showed that more positive outgroup contact predicted youths' frequentation of districts with a higher prevalence of outgroup members (*b* = 5.96, *p* = .008). By contrast, positive ingroup contact was not related to the district use (*b* = −0.45, *p* = .735). Discomfort was not associated with the outgroup prevalence of the district that participants frequented shortly after the interaction. Figure 3 depicts the interaction effect.

**FIGURE 4:**
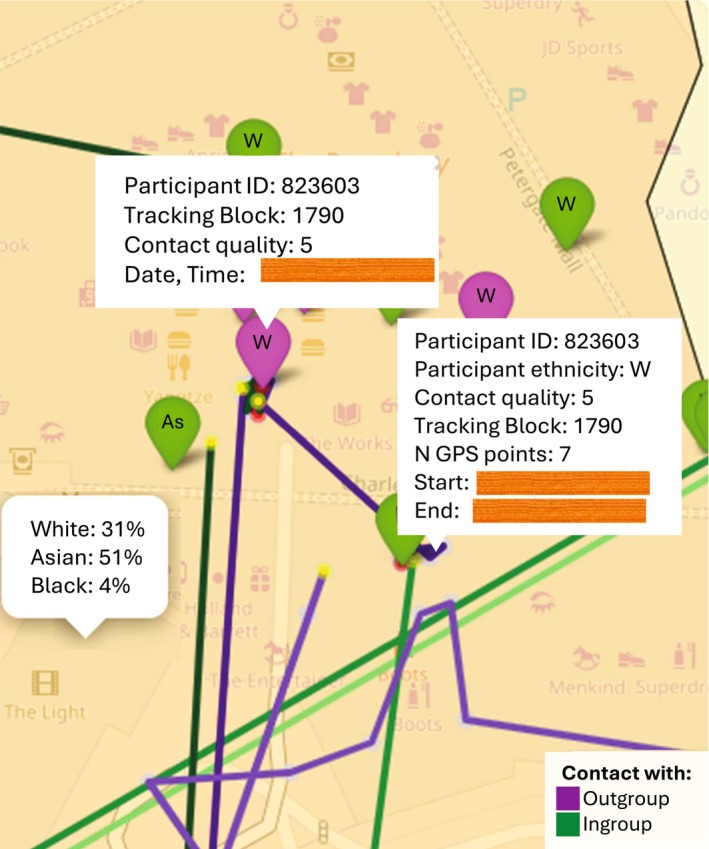
A qualitative example of the Study 2 findings in the Bradford map. Date and time information is blurred to protect the participant's anonymity.

Figure 4 depicts a screenshot from the Bradford .*html* map exemplifying the main result. It highlights an intergroup contact (the purple marker) of a White participant with a Pakistani friend during a very positive social event at the Broadway Bradford shopping centre. After the contact, the GPS records (the purple track line) showed that the participant remained in the Asian‐majority districts (orange‐shaded area) where the shopping area is located for approximately 7 min after the interaction was reported.

### Sensitivity analyses

To assess the robustness of the findings, we conducted sensitivity analyses for both studies (see the Appendix S1). Specifically, we re‐estimated the final models by: (1) including the tracking blocks that exceeded the 4‐h duration threshold, (2) including the negative contacts, and (3) excluding tracking blocks shorter than 1 min (for Study 2). Results remained consistent across these models.

In addition, to address the limitations related to potential confounding factors and the relatively small sample sizes, we conducted pooled analyses across the two studies, while also accounting extensively for contextual confounds in the interaction characteristics (i.e., contact situation, relation, intimacy, formality, duration and age, gender, and number of interaction partners) and the outgroup prevalence of participants' home district. The results replicated the findings of the separate studies. A sensitivity power analysis confirmed that the pooled model had a statistical power of 80% to detect effects of only medium size and above. Importantly, the observed effects exceeded this lower threshold, supporting the appropriateness of the statistical approach alongside the robustness and reliability of the findings.

## GENERAL DISCUSSION

The present research included two studies investigating the reciprocal dynamics between socio‐spatial segregation and intergroup contact among 16–18 years old youth in two divided contexts: Belfast, Northern Ireland – marked by the conflict between Catholics and Protestants – and Bradford, England – a site of interethnic tensions among Asian, White, and Black communities. Adopting an innovative methodology that combined ecological momentary assessment (EMA) of everyday intergroup contact (Keil et al., 2020) with the real‐time GPS tracking and GIS analysis, the studies respond to calls for dynamic, context‐specific methods to explore intergroup contact (Hinds et al., 2022; Paolini, Harwood, et al., 2024). This integrative approach bridges human geography and social psychology, providing novel insights into: (a) how residential segregation limits the opportunity for intergroup contact and (b) whether the quality of previous intergroup contact may influence the subsequent near‐time use of more diverse outgroup spaces, ultimately countering the behavioural segregation in everyday activity space (Liao et al., 2025).

### Residential segregation on intergroup contact

Findings from Study 2 in Bradford suggested that interethnic contact was more likely to occur in urban areas with a higher proportion of outgroup members, supporting the idea that residential mixing can facilitate opportunities for intergroup contact (Schmid et al., 2014; Van Der Laan Bouma‐Doff, 2007). However, neighbourhood diversity did not influence the *quality* of interethnic contact. That is, while residential mixing appeared to increase the likelihood of intergroup encounters, it did not necessarily promote more positive or comfortable interactions. These findings allow speculating that urban spaces may translate into more frequent – but not necessarily more positive – social interactions, a key question posted in human geography (Cagney et al., 2020; Liao et al., 2025). This distinction is crucial, as the finding that neighbourhood diversity did not enhance the quality of intergroup contact allows integrating the mixed literature findings that residential mixing might even lead to threatening intergroup contact and negative attitudes (Dixon et al., 2023; Kotzur & Wagner, 2021; van Kempen & Bolt, 2012), as also shown by Study 1.

Belfast Study 1 showed that intergroup interactions were more uncomfortable than ingroup ones and that greater outgroup presence in the district where the interaction took place was associated with less comfortable intergroup contact. However, it found that higher outgroup prevalence in a given district did not significantly influence the quantity of contact between Catholic and Protestant communities. These findings align with previous findings, which highlighted avoidance and anxiety in the use of outgroup spaces in Belfast (Davies et al., 2019; Dixon et al., 2020, 2022), and can be interpreted through the lens of the Boundary Transgression Model (Dixon et al., 2025). This model suggests that desegregation between historically separated groups and spaces represents a symbolic and material boundary transgression, rupturing the established socio‐spatial order and norms of separation. Such desegregation may trigger intergroup threat and negative attitudes, sustaining micro‐ecological processes of resegregation (Bettencourt et al., 2019) – such as gated communities – intended as defensive efforts to protect ingroup spaces and preserve the existing socio‐spatial structure from the perceived threat of boundary blurring.

Taken together, the two studies and their divergence highlight the role of context‐specific factors in shaping how residential segregation shapes intergroup contact. In Bradford, where relations are characterized by more diffuse interethnic tensions (Miah et al., 2020), neighbourhood diversity and mixing might create opportunities for intergroup contact. By contrast, the Belfast enduring legacy of conflict and norms of segregation (Murtagh et al., 2024) might enforce mental and concrete barriers, inhibiting intergroup interaction through both psychological and spatial mechanisms, ultimately facilitating avoidance and fostering discomfort when transgressing socio‐spatial group boundaries.

### Intergroup contact on activity space segregation

Both studies provided replicated evidence that more positive everyday intergroup contact was associated with youths' near‐time use of spaces with a higher outgroup presence. This finding advances understanding of how microlevel social experiences translate into observable spatial behaviours. The results align with and extend previous work linking self‐reported positive contact with the willingness to use outgroup spaces and time spent in outgroup spaces using GPS mobility data (Dixon et al., 2020) and show that negative contact fosters a generalized outgroup avoidance (Meleady & Forder, 2019). Uniquely, the present research integrates ecological experiences of intergroup contact using EMA (Keil et al., 2020) with near‐time mobility behaviours derived from GPS tracking and GIS analysis. This methodological approach moves beyond the traditional focus on attitude change in contact research, providing compelling evidence that the beneficial effects of positive intergroup contact might foster a tangible behavioural shift – at least in the immediate, short‐term timeframe – (cfr., Grady et al., 2023; Schäfer et al., 2022; Turner & West, 2012), specifically in the use of outgroup spaces, with relevant applied implications for achieving socio‐spatial desegregation.

The findings suggest that positive interactions with outgroup members may encourage people to use outgroup spaces, at least in the short term. Such positive contact could initiate a virtuous circle, promoting micro‐ecological behavioural engagement in outgroup spaces, which contributes to socio‐spatial mixing and desegregation (Dixon et al., 2025). The findings specifically highlight micro‐ecological behaviours, as the research focused on mobility occurring in the near‐time following intergroup contact (i.e., from min up to 4 h or a day), capturing interactions within ordinary and everyday practices and routines (Bettencourt et al., 2019; Dixon et al., 2008). For instance, participants were more likely to linger in outgroup‐dense areas, such as walking in a religious outgroup neighbourhood (Figure 2) or remaining in a shopping area located in an ethnic outgroup district (Figure 4) following a positive intergroup encounter.

### Limitations and Future Directions

Future research should address some of the conceptual and methodological limitations. The current work did not address the psychosocial processes driving the effects of intergroup contact on spatial behaviours. It could be, for example, that experiencing positive contact leads to more favourable intergroup attitudes (Dovidio et al., 2003) that generalize to perceptions towards outgroup spaces (Bonam et al., 2016; Essien & Rohmann, 2024) promoting their use. Another potential mechanism is that intergroup contact could reduce perceived threats to personal safety, ultimately promoting the use of outgroup space. Studies from Northern Ireland and South Africa have shown that positive intergroup contact has the potential to lessen anticipated threat to physical safety, in turn, predicting willingness to use outgroup space, the actual time spent in outgroup areas (Dixon et al., 2020), as well as micro‐ecological processes of resegregation (like building a fence around one's own house or changing mobility habits) and support policies for desegregation (Dixon et al., 2023). Future research should directly test these mechanisms, possibly adopting a methodology that allows for a clear understanding of the precise, time‐contingent information on participants' motivations for or activities following each contact.

The repercussions of negative contact on spatial mobility and resegregation dynamics should also be examined. Although only a few participants reported negative contact – consistent with findings showing that positive contact is more common than negative (Graf et al., 2014; Keil et al., 2020) – negative contact may have a stronger impact on the use of space (Paolini, Gibbs, et al., 2024).

From a methodological perspective, the EMA app recorded only when participants reported an interaction, which may not always be when it actually occurred. This could introduce random noise in the association between contact and subsequent mobility, especially in potential cases where participants would log a contact with a substantial delay, resulting in a temporal misalignment between the interaction and subsequent movements. Future EMA studies should control this risk by allowing participants to record the exact contact timing. Besides, a key limitation concerns the number of participants, the volume of EMA‐reported contacts, and the density of GPS data. Separately, the studies had relatively small samples and numbers of reported contacts, which may limit the reliability of the findings. However, as further detailed in the Appendix S1, the replication of the results across the two studies and the pooled analysis, previous statistical simulations, the sensitivity power analysis, and empirical precedents effectively mitigate concerns about sample size and statistical power, strongly supporting the robustness of the findings.

In addition, the low density of GPS tracking is reflected in several short GPS tracking blocks (e.g., < 1 min): the short GPS segments may simply capture the location of the interaction itself rather than genuine post‐contact mobility. Consequently, it is possible that our findings partly reflect positive contacts occurring within outgroup‐dense areas, rather than positive contact prompting subsequent use of outgroup urban districts. We adopted several strategies to mitigate these concerns. First, preliminary analyses in both studies showed that district‐level outgroup prevalence did not predict the valence of intergroup contact, ruling out the alternative explanation that positive contacts were more likely to occur in districts with higher outgroup prevalence. Second, following similar approaches (Dixon et al., 2020), supplementary analyses for Study 2 showed that the results remained robust even after excluding tracking blocks shorter than 1 min. Third, while some GPS tracking blocks were indeed brief, half of the blocks used in the main analyses lasted longer than the median duration (~1 min), extending up to 4 h, hence mitigating the risk that the dependent variable could reflect instant locations of where the contacts occurred. Therefore, these methodological controls substantially diminish the methodological limitation related to the volume and duration of GPS tracking blocks.

## CONCLUSION

The research provides compelling evidence about the interplay between everyday intergroup contact and socio‐spatial segregation, serving as a bridge between social psychology's understanding of intergroup contact with human geography's insights into spatial dynamics and urban life. The theoretical implications are twofold: on the one hand, the present research contributes to the human geography literature by highlighting the bidirectional link between urban mixing and intergroup interactions; on the other hand, it advances intergroup contact theory by showing that the benefits of positive contact extend beyond attitudinal shifts to impact mobility behaviours. From a methodological standpoint, the research introduces a cutting‐edge approach to capturing everyday interactions in real‐world contexts integrated with GPS‐based assessment of spatial mobility, offering a concrete tool for interdisciplinary research addressing the spatial dimensions of psychosocial phenomena.

These insights have potential applied implications. Urban planning and community initiatives aimed at fostering socio‐spatial desegregation should therefore consider strategies that actively promote inclusive public spaces fostering opportunities for positive intergroup encounters among youth. By instigating a virtuous circle – where positive contact encourages use of outgroup space, which in turn can foster further positive interactions – even everyday encounters can contribute to breaking down societal divides and fostering more integrated urban environments for the younger generation.

## AUTHOR CONTRIBUTIONS

**Marco Marinucci:** Conceptualization; methodology; writing – original draft; visualization; formal analysis; data curation; validation; writing – review and editing. **Christoph Daniel Schaefer:** Conceptualization; writing – review and editing; methodology; validation. **Pier‐Luc Dupont:** Conceptualization; writing – review and editing; investigation; methodology. **David Manley:** Conceptualization; writing – review and editing; funding acquisition; methodology; project administration. **Laura K. Taylor:** Conceptualization; funding acquisition; writing – review and editing; methodology; project administration. **Shelley McKeown Jones:** Conceptualization; funding acquisition; writing – review and editing; project administration; supervision; resources; investigation; data curation.

## CONFLICT OF INTEREST STATEMENT

Shelley McKeown Jones, Co‐Editor‐in‐Chief of the British Journal of Social Psychology, is a co‐author on this manuscript. In accordance with journal policy, SMJ had no role in the editorial review or decision‐making process for this submission; the manuscript was handled independently by another editor. The other authors report no conflicts of interest.

## Supporting information


Appendix S1.

## Data Availability

All research data, analysis codes, and Appendix S1 are available via this OSF link (https://osf.io/vt4u8/overview?view_only=467e3c2034414f2a90c2b0b0e19744e2).

## Footnotes

^1^
The research is part of a larger project on intergroup contact among youth (see McKeown et al., 2024). In the current research subsamples of participants from the broader project were selected to take part in the EMA studies with GPS recording.

^2^
Coefficients are exp(b) because the mixed‐model regression was fitted specifying a gamma distribution with a log‐link function to account for the strong right‐skewness of the dependent variable. The linear function yielded convergence issues.
