# Supplementary material for: Desegregating spaces: The interplay between ecological intergroup contact and GPS‐traced spatial segregation among youth in two UK cities
Source: Br J Soc Psychol. 2026 Jan 5;65(1):e70043. doi: 10.1111/bjso.70043 (PMC12770809; doi:10.1111/bjso.70043)
Supplement: Supplementary file 1 — Appendix S1. [file BJSO-65-0-s001.docx]

**Supplemental Materials**

This file reports the following:

1. Details of the response categories in the *Contact Logger* app;
2. Sensitivity analyses for the main results alongside alternative analyses:
   1. Including tracking blocks exceeding a timespan of 4 hours,
   2. Including both positive and negative contact as predictors,
   3. Excluding tracking blocks lasting less than 1 minute
3. The pooled analyses based on merged Study 1 and 2 data
4. A sensitivity power analysis for the pooled analyses
5. Supplementary references
6. **Details about the response scales and categories describing social interactions**

This section details the response scale and categories to assess social interactions via the *Contact Logger* app. Participants recorded detailed information about each interaction, including the setting, situation, duration, and level of formality. They also indicated whether the interaction occurred with an individual or a group. Additionally, they reported the interaction partner’s ethnicity/community background, gender, religion, age group, and perceived group typicality, as well as the nature of their relationship. Finally, participants rated both the level of anxiety and the quality of the interaction.

Participants could turn on/off the GPS tracking system operating in the background by tapping on the icon circled in Figure 1S.

*
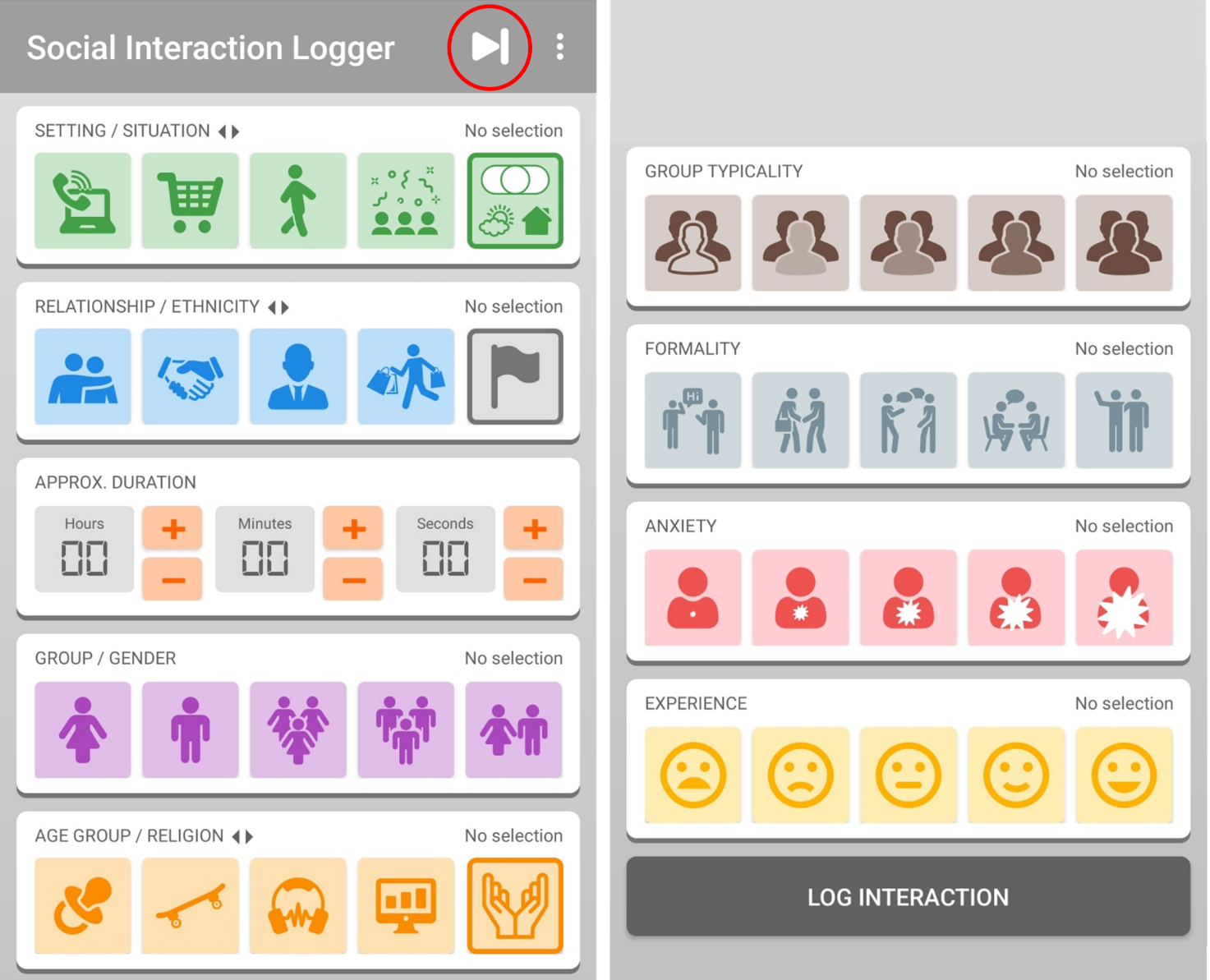
Figure 1S*. Interface of the *Contact Logger* app

Below are the detailed response options for each construct.

**Setting.** 1) Indoor, 2) Outdoor

**Situation.** 1) Online – Phone, 2) Shopping, 3) Walking, 4) Social event, 5) Workplace, 6) Private transport, 7) Public transport, 8) Home, 9) Food & drink outlet, 10) Club – Hobby activity, 11) Place of worship, 12) Other (specify).

**Relationship.** 1) Friend, 2) Acquaintance, 3) Colleague, 4) Customer – Client, 5) Stranger, 6) Service clerk, 7) Neighbour, 8) Partner – Spouse, 9) Relative, 10) Other (specify).

**Ethnicity.** 1) White British – English – Northern Irish – Scottish – Welsh, 2) White Irish, 3) Irish Traveller, 4) Eastern European, 5) Roma, 6) Indian, 7) Pakistani, 8) Bangladeshi, 9) Chinese, 10) Filipino, 11) Black African, 12) Black Caribbean, 13) Mixed Ethnicity, 14) Other, 15) Mixed group, 16) Not sure.

For conciseness, we recoded the 16 ethnic groups into 5 broad categories as follows, according to the highest level of ethnic categorization based on the 2021 England and Wales CENSUS (<https://www.ons.gov.uk/peoplepopulationandcommunity/culturalidentity/ethnicity/bulletins/ethnicgroupenglandandwales/census2021>): 1) White (from 1 to 5), 2) Asian (from 6 to 10), 3) Black (11, 12), 4) Mixed – unsure, 5) other.

**Duration.** Approximate duration of the interactions in hours, minutes, and seconds.

**Group/Gender.** 1) Single female, 2) Single male, 3) Group (all female), 4) Group (all male), 5) Group (mixed gender).

**Age group.** 1) Baby – small child, 2) Child, 3) Young person, 4) Adult, 5) Older adult, 6) Mixed age group.

**Religion.** 1) Protestant, 2) Catholic, 3) Buddhist, 4) Hindu, 5) Jewish, 6) Muslim, 7) Sikh, 8) Other, 9) Mixed group, 10) Not sure.

**Group typicality.** 1-5 Likert scale (1 = not typical at all, 5 = very typical).

**Formality.** 1-5 Likert scale (1 = very casual, 5 = very meaningful).

**Anxiety.** 1-5 Likert scale (1 = not at all uncomfortable, 5 = very uncomfortable)

**Quality.** 1-5 Likert scale (1 = very negative, 3 = neutral, 5 = very positive).

After logging the interaction, participants specified the exact location where the contact occurred, as depicted in Figure 2S.


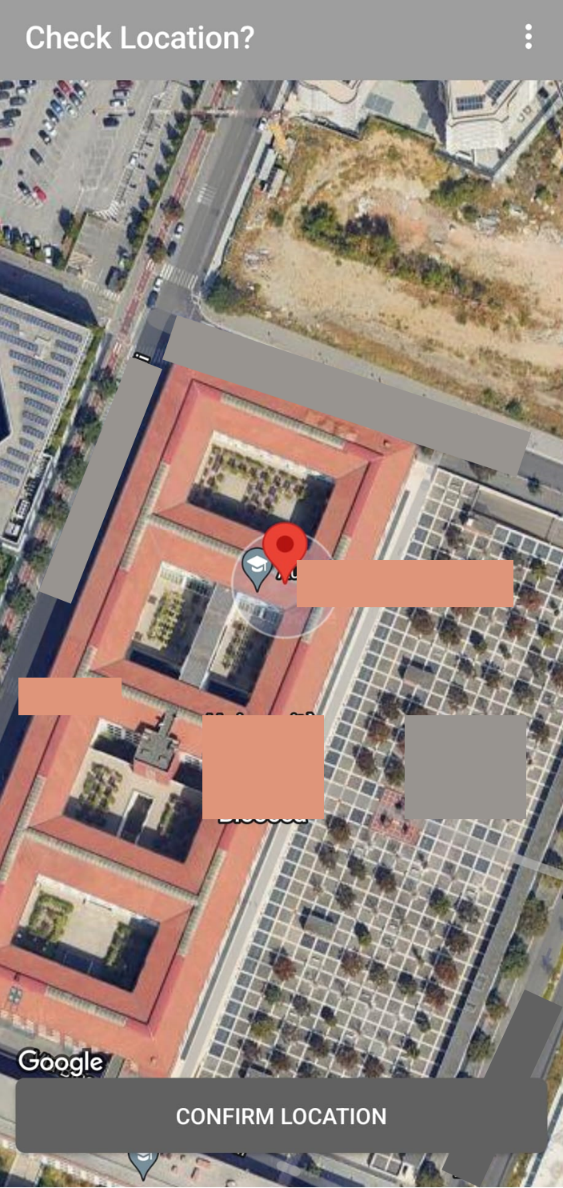
*Figure 2S.* Interaction location

*Note.* Identifiable information is blurred.

1. **Sensitivity analyses**

**Study 1**

Results from additional models including the GPS tracking blocks exceeding 4 hours (Model 1.1) and the negative outgroup interaction alongside the positive ones (Model 1.2), were consistent with the main models presented in the manuscript: the models highlight a positive significant interaction between contact quality and the group with the interaction partner, indicating that more positive contact with outgroup members predicted the subsequent use of urban spaces with higher outgroup members’ proportion. (Tables 1S). Simple slope analyses highlighted that the valence of the previous interaction did not affect mobility following ingroup contact (Model 1.1: b = -2.60, *p* = .529; Model 1.2: b = -3.47, *p* = .413). In contrast, more positive outgroup contact was associated with youth frequentation of districts with higher outgroup prevalence after the interaction (Model 1.1: b = 17.49, *p* = .017) - although the slope of Model 1.2 was only marginally significant (b = 13.11, *p* = .065). Discomfort with either group was not associated with subsequent mobility. In Study 1, it was not possible to test an additional model excluding tracking blocks shorter than 1 minute because doing so would have resulted in retaining only 22 observations, an extremely low and insufficient sample size.

**Study 2**

Results from the additional models, including the GPS tracking blocks exceeding 4 hours (Model 2.1), the negative outgroup interaction alongside the positive ones (Model 2.2), and excluding tracking blocks shorter than 1 minute (Model 2.3), remained consistent with the main models presented in the manuscript. The interaction effect of contact quality and the group of the interaction partner was positive and significant in the three models (Table 2S). Simple slope analyses showed that contact valence did not influence youth’s presence in districts with higher outgroup prevalence, following ingroup contact (Model 2.1: b = -0.66, *p* = .661; Model 2.2: b = -0.83, *p* = .444; Model 2.3: b = 0.16, *p* = 949). In contrast, more positive outgroup interactions predicted youth frequentation of districts with higher outgroup prevalence after the interaction (Model 2.1: b = 5.71, *p* = .014; Model 2.2: b = 2.63, *p* = .080; Model 2.3: b = 8.08, *p* =.011) – although the slope of Model 2.2 was only marginally significant. Discomfort with either group did not predict youth mobility after the interaction.

*Table 1S.* Positive and negative interactions as predictors – Study 1

|  | Model 1.1: add tracking blocks > 4 hours | | | |  | Model 1.2: add the negative interaction | | | |
| --- | --- | --- | --- | --- | --- | --- | --- | --- | --- |
| *Predictors* | *Estimates* | *95%CI* | *p* | *Cohen’s d* |  | *Estimates* | *95%CI* | *p* | *Cohen’s d* |
| Fixed Effects |  |  |  |  |  |  |  |  |  |
| (Intercept) | 44.66 | 1.38 – 87.94 | **.*043*** |  |  | 48.92 | 4.83 – 93.00 | **.*030*** |  |
| Discomfort | 4.75 | -4.68 – 14.17 | .318 | 0.28 |  | 4.28 | -5.37 – 13.93 | .379 | 0.24 |
| Group | -100.03 | -183.55 – -16.51 | **.*020*** | -0.64 |  | -86.53 | -171.40 – -1.65 | **.*046*** | -0.54 |
| Valence | -2.60 | -10.81 – 5.60 | 0.528 | -0.18 |  | -3.47 | -11.86 – 4.92 | .412 | -0.24 |
| Discomfort×Group | 6.87 | -8.32 – 22.07 | 0.369 | 0.24 |  | 8.81 | -6.67 – 24.30 | .260 | 0.30 |
| Valence×Group | 20.03 | 3.99 – 36.08 | **.*015*** | 0.68 |  | 16.57 | 0.44 – 32.71 | **.*044*** | 0.55 |
| Random Effects | | | | |  |  |  |  |  |
| σ^2^ | 139.51 | | | |  | 149.24 | | | |
| τ_00_: hour/day/subject  day/subject  subject | 199.37  15.40  568.25 | | | |  | 213.67  9.85  549.62 | | | |
| N Observation  N (hour/day/subject) | 73  (18 / 6 / 14) | | | |  | 74  (18 / 6 / 14) | | | |

*Note.* σ^2^ = residual variance; τ_00_ = random intercept variance

*Table 2S.* Sensitivity analyses – Study 2

|  | Model 2.1: add tracking blocks > 4 hours | | | |  | Model 2.2: add negative interactions | | | | | | |  | | | Model 2.3: exclude tracking blocks < 1 minute | | | | | | | | | |  |
| --- | --- | --- | --- | --- | --- | --- | --- | --- | --- | --- | --- | --- | --- | --- | --- | --- | --- | --- | --- | --- | --- | --- | --- | --- | --- | --- |
| *Predictors* | *Estimates* | *95%CI* | *p* | *Cohen’s d* |  | *Estimates* | | *95%CI* | | *p* | | *Cohen’s d* | | |  | | | *Estimates* | | *95%CI* | | *p* | | *Cohen’s d* | | |
| Fixed Effects |  |  |  |  |  | |  | |  | |  | | |  | | |  | |  | |  | |  | |  |  |
| (Intercept) | 57.56 | 41.39 – 73.73 | **<.*001*** |  | 57.48 | | 43.16 – 71.80 | | **<.*001*** | |  | | |  | | | 63.09 | | 38.96 – 87.21 | | **<.001** | |  | |  |  |
| Discomfort | -3.29 | -6.78 – 0.20 | .064 | -0.34 | -2.01 | | -5.27 – 1.24 | | .224 | | -0.21 | | |  | | | -8.16 | | -12.44 – -3.89 | | **<.001** | | -1.23 | |  |  |
| Group | -22.98 | -47.61 – 1.65 | .067 | -0.35 | -11.52 | | -30.42 – 7.39 | | .231 | | -0.24 | | |  | | | -30.48 | | -68.44 – 7.48 | | .114 | | -0.44 | |  |  |
| Valence | -0.66 | -3.63 – 2.30 | .661 | -0.09 | -0.95 | | -3.38 – 1.47 | | .441 | | -0.16 | | |  | | | 0.16 | | -4.69 – 5.00 | | .949 | | -0.02 | |  |  |
| Discomfort×Group | 0.59 | -6.40 – 7.58 | .868 | 0.03 | -0.21 | | -6.59 – 6.18 | | .949 | | 0.01 | | |  | | | 1.83 | | -6.84 – 10.49 | | .677 | | 0.13 | |  |  |
| Valence×Group | 6.37 | 1.30 – 11.45 | **.*014*** | 0.47 | 3.86 | | 0.13 – 7.59 | | **.*043*** | | 0.41 | | |  | | | 7.93 | | 0.33 – 15.52 | | **.*041*** | | 0.60 | |  |  |
| Random Effects | | | | | | | | | | | | | | | | | | | | | | | | | |  |
| σ^2^ | 56.49 | | | |  | 55.83 | | | | | | |  | | | 46.51 | | | | | | | | | |  |
| τ_00_: hour/day/subject  day/subject  subject | 206.60  142.69  394.76 | | | |  | 221.28  146.04  382.99 | | | | | | |  | | | 219.19  88.25  357.93 | | | | | | | | | |  |
| N Observation  N (hour/day/subject) | 229  (19 / 6 / 30) | | | |  | 234  (19 / 6 / 30) | | | | | | |  | | | 115  (15 / 6 / 24) | | | | | | | | | |  |

*Note.* σ^2^ = residual variance; τ_00_ = random intercept variance

1. **Pooled analyses**

This section presents pooled data analyses for Studies 1 and 2 to overcome power and sample size limitations when analysing each study separately; Study 1 main results were based on 70 observations, and Study 2 on 219 observations. We reasoned that analysing the pooled data might strengthen the robustness and reliability of the findings. We adopted the same analytical approach as the main analyses of separate studies.

First, comparison of null models showed that clustering the random intercepts of the GPS tracking data as hours within day within participants produced a better fitting model compared to clustering days within participants (Δχ^2^(1) = 90.56, *p <* .001) or participants only (Δχ^2^(2) = 115.13, *p <*.001). We then conducted the main analyses with positive contacts only and GPS tracking blocks that did not exceed 4 hours, following the same analytical strategy adopted for the main analyses reported in the manuscript. Additionally, we tested a model that accounted for the outgroup prevalence of the participants’ home district as well as potential contextual confounds related to the specific interaction, namely the contact situation, the relation with, age, and gender of the interacting partners(s), the intimacy (intimate *vs.* superficial), and the number of interacting partners (whether it occurred with another person or with a group), the duration of the interaction, and its perceived formality (see section 1 for the detailed response categories). These were included as the increase in sample size could compensate for the drop in the number of observations due to missing values in the control variables. The findings replicated those reported in the manuscript (Table 3S). Simple slope analyses showed that more positive outgroup contact was associated with subsequent presence in districts with higher outgroup prevalence (Main Model: b = 6.66, *p* = .004; Control variable model: b = 7.81, *p* = .027) whilst positive ingroup contact did not influence the use of outgroup spaces (Main Model: b = -0.85, *p* = .538; Control variable model: b = -0.64, *p* = .796). Interaction effects from the Main model are depicted in Figure 3S. As in the separate studies, we found that the observed effect did not change when including tracking blocks covering a timespan exceeding 4 hours and negative interactions as predictors. These analyses are not reported here for conciseness but are available in the *pooled analyses* R script on the OSF.

*Table 3S.* Pooled results

|  | Main model | | | | | | | | |  | Control variables | | | | | | |
| --- | --- | --- | --- | --- | --- | --- | --- | --- | --- | --- | --- | --- | --- | --- | --- | --- | --- |
| *Predictors* | *Estimates* | | | *95%CI* | | | *p* | | *Cohen’s d* |  | *Estimates* | | | *95%CI* | | *p* | *Cohen’s d* |
| Fixed Effects |  | | |  | | |  | |  |  |  | | |  | |  |  |
| (Intercept) | 54.99 | | | 40.11 – 69.86 | | | **<.*001*** | |  |  | 43.72 | | | 17.04 – 70.40 | | **.*001*** |  |
| Discomfort | -3.09 | | | -6.36 – 0.17 | | | .063 | | -0.32 |  | -2.98 | | | -7.35 – 1.40 | | .182 | -0.25 |
| Group | -32.86 | | | -58.14 – -7.59 | | | **.*011*** | | -0.44 |  | -35.28 | | | -72.21 – 1.65 | | .061 | -0.35 |
| Valence | -0.85 | | | -3.56 – 1.86 | | | .538 | | -0.12 |  | -0.64 | | | -5.54 – 4.26 | | .796 | -0.05 |
| Discomfort×Group | 3.63 | | | -2.16 – 9.41 | | | .218 | | 0.21 |  | 2.88 | | | -5.89 – 11.66 | | .518 | 0.10 |
| Valence×Group | 7.51 | | | 2.43 – 12.59 | | | **.*004*** | | 0.51 |  | 8.46 | | | 1.01 – 15.91 | | **.*026*** | 0.43 |
| House district  outgroup prevalence |  | | |  | | |  | |  |  | 0.35 | | | 0.16 – 0.53 | | **<.*001*** | 1.28 |
| Contact intimacy |  | | |  | | |  | |  |  | 2.10 | | | -11.23 – 15.43 | | .756 | 0.05 |
| Dyadic/group contact |  | | |  | | |  | |  |  | -6.96 | | | -16.50 – 2.58 | | .152 | -0.23 |
| Situation [leisure time]^a^ |  | | |  | | |  | |  |  | 4.02 | | | -6.53 – 14.57 | | .454 | 0.14 |
| Situation [online]^a^ |  | | |  | | |  | |  |  | -6.96 | | | -17.68 – 3.75 | | .201 | -0.22 |
| Situation [other]^a^ |  | | |  | | |  | |  |  | -4.69 | | | -18.52 – 9.13 | | .504 | -0.12 |
| Situation [social event]^a^ |  | | |  | | |  | |  |  | 3.08 | | | -8.81 – 14.96 | | .610 | 0.09 |
| Situation [transport]^a^ |  | | |  | | |  | |  |  | -8.18 | | | -20.21 – 3.86 | | .182 | -0.22 |
| Situation [walk]^a^ |  | | |  | | |  | |  |  | 5.17 | | | -5.84 – 16.19 | | .355 | 0.15 |
| Situation [work]^a^ |  | | |  | | |  | |  |  | 3.09 | | | -8.92 – 15.10 | | .612 | 0.09 |
| Situation [worship]^a^ |  | | |  | | |  | |  |  | -11.59 | | | -37.11 – 13.92 | | .371 | -0.16 |
| Duration |  | | |  | | |  | |  |  | 0.00 | | | -0.00 – 0.00 | | .913 | 0.02 |
| Formality |  | | |  | | |  | |  |  | 0.66 | | | -1.57 – 2.89 | | .558 | 0.09 |
| Relation [client/customer]^b^ |  | | |  | | |  | |  |  | -9.41 | | | -25.43 – 6.60 | | .248 | -0.18 |
| Relation [colleague]^b^ |  | | |  | | |  | |  |  | -3.03 | | | -16.88 – 10.82 | | .667 | -0.09 |
| Relation [friend]^b^ |  | | |  | | |  | |  |  | -5.83 | | | -18.38 – 6.72 | | .361 | -0.14 |
| Relation [partner]^b^ |  | | |  | | |  | |  |  | -12.70 | | | -28.24 – 2.83 | | .108 | -0.25 |
| Relation [service clerk]^b^ |  | | |  | | |  | |  |  | 5.10 | | | -6.61 – 16.81 | | .392 | 0.17 |
| Relation [stranger]^b^ |  | | |  | | |  | |  |  | -1.63 | | | -11.92 – 8.67 | | .755 | -0.06 |
| Age [child]^c^ |  | | |  | | |  | |  |  | 4.29 | | | -20.17 – 28.74 | | .730 | 0.06 |
| Age [older adult]^c^ |  | | |  | | |  | |  |  | 2.50 | | | -13.85 – 18.85 | | .763 | 0.05 |
| Age [teenager]^c^ |  | | |  | | |  | |  |  | 2.54 | | | -3.70 – 8.78 | | .424 | 0.16 |
| Gender [male]^d^ |  | | |  | | |  | |  |  | 0.58 | | | -3.92 – 5.08 | | .799 | 0.05 |
| Random Effects | |  |  | |  |  | |  | | | |  |  | |  |  |  |
| σ^2^ | 56.21 | | | | | | | | |  | 80.41 | | | | | | |
| τ_00_: hour/day/subject  day/subject  subject | 292.62  55.13  461.20 | | | | | | | | |  | 232.67  58.74  297.79 | | | | | | |
| N Observation  N (hour/day/subject) | 289  (19 / 12 / 44) | | | | | | | | |  | 224^x^  (19 / 12 / 40) | | | | | | |

*Note.* σ^2^ = residual variance; τ_00_ = random intercept variance; ^x^ the drop in sample size is due to missing values in the control variables; ^a^ = reference category: home; ^b^ = reference category: acquaintance; ^c^ = reference category: adult; ^d^ = reference category: female.

*Figure 3S.* Interaction effect of contact positivity by group – pooled data


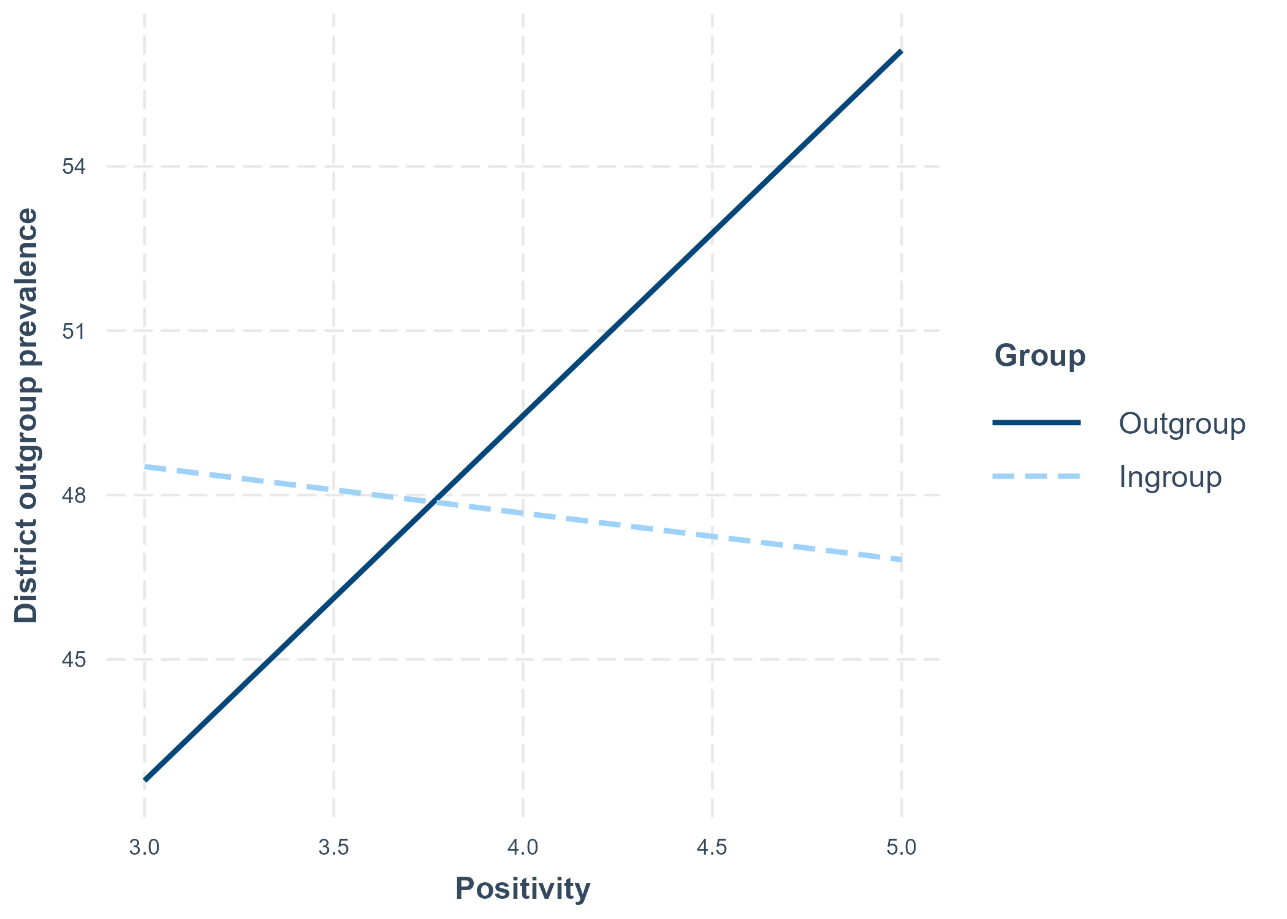


1. **Sensitivity power analysis**

We conducted a simulation-based sensitivity power analysis for mixed models using the *simr* package (Green & MacLeod, 2016). Based on 1000 Monte Carlo simulations (α = 0.05) the analysis indicated that the sample size achieved in the main model of the pooled data set could detect, with 80% power, medium and above effect sizes (Cohen’s d >= 0.50). The effect size for the valence*group interaction with the achieved model size (Cohen’s d = 0.51) is higher than the one detectable with 80% power, meaning that the pooled analysis was well-powered. Besides, the results from the sensitivity power analysis align with previous statistical simulation studies from EMA designs, which converged in indicating that power to detect effects of medium magnitude stabilises above 80% with approximately 45 participants providing 5–10 observations each (Scherbaum & Ferreter, 2009; Oleson et al., 2021; van de Matt et al., 2024). Indeed, a recent systematic review on EMA/ESM studies about intergroup discrimination (Sladek et al., 2025) highlights that many published intensive EMA/ESM studies in this domain rely on comparable sample sizes, with roughly 20% of them including around 50 participants or fewer.

In conclusion, while we acknowledge that the separate studies are limited in size, the replication of the results across two studies, supported by the pooled analysis, convergent statistical simulations, sensitivity power checks, and empirical precedent, effectively mitigates concerns about sample size and statistical power, strongly supporting the robustness and reliability of the reported findings.

1. **Supplementary references**

Oleson, J. J., Jones, M. A., Jorgensen, E. J., & Wu, Y. H. (2022). Statistical Considerations for Analyzing Ecological Momentary Assessment Data. Journal of speech, language, and hearing research : JSLHR, 65(1), 344–360. https://doi.org/10.1044/2021_JSLHR-21-00081

Scherbaum, C. A., & Ferreter, J. M. (2008). Estimating Statistical Power and Required Sample Sizes for Organizational Research Using Multilevel Modeling. *Organizational Research Methods, 12*(2), 347-367. https://doi.org/10.1177/1094428107308906

Sladek, M. R., Martinez-Fuentes, S., Wantchekon, K. A., Aguilar, G., & Umaña-Taylor, A. J. (2025). Ethnic–racial discrimination, identity, and out-group contact in context: A systematic review of daily process studies. *Cultural Diversity & Ethnic Minority Psychology.* Advance online publication. https://dx.doi.org/10.1037/cdp0000735

Van de Maat, R., Lataster, J., & Verboon, P. (2024). Minimum Required Sample Size for Modelling Daily Cyclic Patterns in Ecological Momentary Assessment Data. *Methodology, 20*(4), 265-282. https://doi.org/10.5964/meth.11399
